# Exploring the potential of the metaverse medical paradigm in drug addiction treatment: a preliminary discussion and future prospects

**DOI:** 10.1136/gpsych-2023-101258

**Published:** 2023-12-14

**Authors:** Longtao Yang, Lijie Zhang, Wenhan Yang, Fei Tang, Yanyao Du, Jun Liu

**Affiliations:** 1 Department of Radiology, The Second Xiangya Hospital, Central South University, Changsha, Hunan, China; 2 Clinical Research Center for Medical Imaging in Hunan Province, Changsha, Hunan, China; 3 Department of Radiology Quality Control Center in Hunan Province, Changsha, Hunan, China

**Keywords:** Behavior Observation Techniques, Cognitive Behavioral Therapy, Virtual Reality Exposure Therapy, Cognitive Neuroscience, Behavior and Behavior Mechanisms

## Introduction

Drug addiction is a chronic and recurrent encephalopathy characterised by impulsive behaviour, spiritual cravings, psychological distortion and physical damage.^1^ According to the role of molecular biology mechanisms on the central nervous system, addictive substances can be classified as inhibitors (eg, opioids, etc), stimulants (eg, methamphetamine (MA), nicotine, cocaine, etc) and hallucinogens (eg, cannabis, etc).^1^ As published by the World Drug Report 2022, over 284 million individuals aged 15–64 worldwide have reportedly abused drugs in the past 12 months, emphasising the international challenge of effective detox treatment. The detoxification process consists of three phases: physiological recovery for the remission of withdrawal symptoms, psychological rehabilitation for the elimination of mental dependence, and social function restoration for the return to life, with the second phase being the most significant challenge. A sustained high level of drug craving often leads to unsuccessful psychological rehabilitation. Current methods of psychological intervention generally include compulsory detention management, cognitive behavioural therapy (CBT), repetitive transcranial magnetic stimulation (rTMS) and deep brain stimulation (DBS).^2^ However, often drug cravings are not effectively controlled.^3^ For example, the relapse rate of individuals addicted to opioid drugs like heroin could exceed 95% within 6 months post-detox treatment.^4^ Therefore, there is an urgent need to consider treatment effects during psychological rehabilitation. The integration of multiple schemes and novel interventions for drug treatment is emerging as a key research topic in this field.

**Figure 1:**
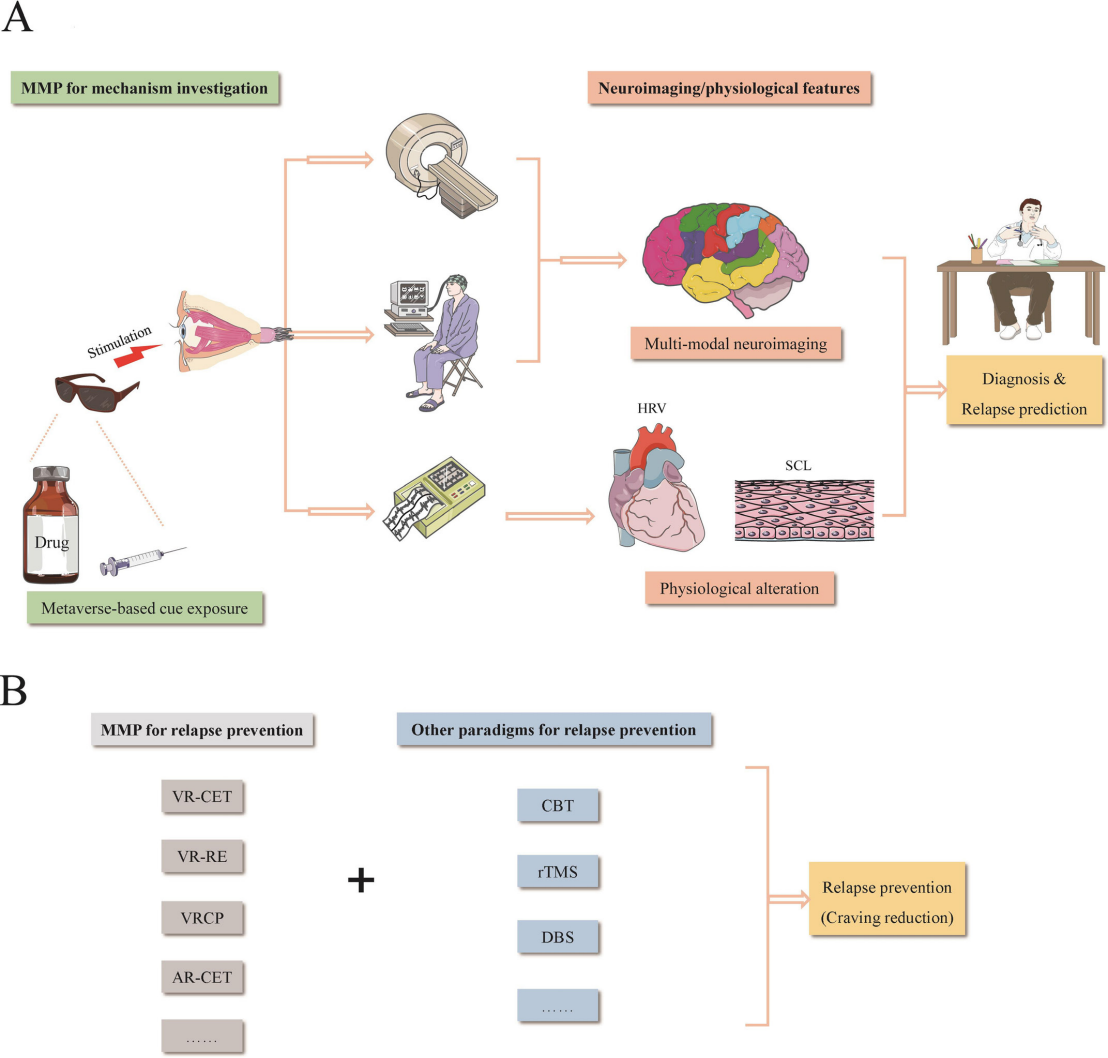
Schematic illustration of the role of metaverse medical paradigms in biological mechanism exploration (A) and relapse prevention (B) of drug addiction. AR-CET, augmented reality-cue exposure therapy; CBT, cognitive behavioural therapy; DBS, deep brain stimulation; HRV, heart rate variability; MMP, metaverse medical paradigm; rTMS, repetitive transcranial magnetic stimulation; SCL, skin conductance level; VR, virtual reality; VR-CET, VR-cue exposure therapy; VRCP, VR counterconditioning producer; VR-RE, VR-retrieval-extinction paradigm. Figure 1(A) is adapted from Servier Medical Art with publication permission.

In recent years, digital health has rapidly advanced, with a focus on improving the efficiency and quality of healthcare services, including diagnosis, monitoring, treatment and data management, through digital information communication. Metaverse, also known as the three-dimensional internet, is a broader concept involving virtual multidimensional spaces that foster social interactions, user-generated content and a persistent environment across various domains, such as economy, ecology and the medical industry. Metaverse is a remarkable accomplishment in cutting-edge technologies that can be divided into four essential categories: mixed reality (MR), extended reality, virtual reality (VR) and augmented reality (AR).^5^ Of these, VR especially enables individuals to immerse themselves in and interact with a virtual world.^5^ Unlike VR, AR typically inserts created images into the experiencer’s coexisting environment via a smartphone or tablet.^5^ Therefore, the metaverse paradigm applied in the medical field can be defined as the Internet of Things promoted by AR, VR and even MR.^5^ The metaverse medical paradigm (MMP) has been employed in the treatment for mental disorders such as depression, anxiety and autism.^5^ As depicted in figure 1, in the neuropsychiatric field of drug addiction, there are two main application modes of the MMP through visual induction. One mode is for neuroimaging mechanism exploration, mediating the fluctuations of physiological activities through cue exposure stimulation and leading to the identification of potential therapeutic neuro-targets and predictive and diagnostic biomarkers. The other mode is for detox treatment, reconstructing the psychological environment and thereby influencing behaviour and cognition during psychological rehabilitation. Thus, MMP will be able to provide a new perspective for in-depth delving into all aspects of substance use disorder (SUD), covering diagnosis, prediction and treatment. To clarify, while traditional approaches like self-reported drug consumption or urine/hair tests can readily identify SUD, diagnostic neuroimaging biomarkers could facilitate the discovery of predictive and therapeutic markers.

## MMP for neuroimaging mechanism exploration

Cerebral imaging techniques, including functional magnetic resonance imaging (fMRI), electroencephalogram (EEG) and physiological activity measures (including heart rate variability (HRV) and skin conductance level (SCL)), can provide objective indicators for the response to external stimuli. When exposed to substance cues, a VR scene has been more effective in transforming cerebral reactivity than a video or still picture, eliciting higher drug craving scores.^6^ The transition from baseline resting state to MA-related VR-cue conditions revealed a significant difference in the non-linear and time domains of HRV between those with MA addictions and healthy controls and was positively correlated with craving severity.^7^ Neural correlates of this induction involved decreased EEG gamma activity in the dorsolateral prefrontal cortex and medial prefrontal cortex/orbitofrontal cortex, which was responsible for executive control and the reward circuit.^8^ However, there was no association between the brain’s electrophysiological response and self-reported craving.^8^ The evaluation of drug craving via a visual analogue scale is susceptible to reporting and interviewer bias, indicating the necessity of employing more sensitive and objective indexes that represent biological alterations during stimulation experiments, such as autonomic nervous reactivity like SCL.^8^ Interestingly, the arousal of SCL and HRV was used as a physiological marker for craving reactivity.^9^

Presently, due to its protruding shape, a head-mounted device for VR or AR is challenging to match with the head coil of an MRI machine, limiting the discovery of the biological mechanisms of MMP in neuropsychiatric imaging (eg, fMRI, etc). Thus, MMP equipment that is better designed in the future will facilitate the progression of task fMRI for identifying specific neural biomarkers. In addition, beyond comparing the groups with addictions and healthy controls in MMP trials, subgroup analysis of the cerebral features and other physiological measurements among those with addictions undergoing varied psychological interventions (eg, rTMS, MMPs for rehabilitation, DBS, etc) may also be informative in developing novel therapeutic mechanisms and personalising treatment plans. More research in this field is needed, despite the challenges confronting it. Standardised reference guidelines for constructing MMPs are lacking. Moreover, enrolling participants addicted to illegal drugs such as heroin or MA or to emerging substances that are being abused such as etomidate and laughing gas has become even more difficult since the coronavirus disease 2019 (COVID-19) pandemic in China because of the reduction of available subjects, either due to death or loss of follow-up contact.

## MMP for psychological rehabilitation

Relapse is commonly characterised by substance craving. To address this, MMP can play a positive role in drug desensitisation, including drug-related VR-cue exposure therapy (VR-CET), VR-retrieval-extinction paradigm (VR-RE), VR counterconditioning producer (VRCP), and AR-cue exposure therapy (AR-CET). Nicotine-related VR-CET is found to be at least as effective as CBT in reducing drug craving, with significantly lower dropout and relapse rates.^10^ A case report involving six VR-CET sessions over 5 weeks preliminarily showed improved anxiety and decreased craving as well as altered attention bias in the post-treatment phase of alcohol use disorder.^11^ Based on the theory of memory reconsolidation, the efficacy of VR-RE in decreasing cue-induced craving was validated in MA participants.^9^ Following VRCP with the stimulus of an opposite valence, MA subjects exhibited a more substantial decrease in HRV indexes on the non-linear domain and time domain during VR cue exposure, in contrast to VRCP-untreated individuals with addicitions.^7 12^ Moreover, VR-CET combined with mindfulness-based relapse prevention might have critical clinical application.^13^ Vinci *et al* first proposed AR-CET in nicotine cessation, using a smartphone application with smoking-related AR images and neutral AR images, and observed that exposure to the former mediated a higher urge to smoke in a sample of 10 smokers.^14^ This was a groundbreaking trial of applying AR to cue exposure treatment.

However, in the psychosphere of drug addiction, the sample sizes in the varied MMP studies have been relatively small. Sensitivity to MMPs might differ among individuals with SUDs, influenced by factors such as heredity, surrounding environment, and educational background. Thus, increasing the number of subjects will mitigate random errors and improve statistical power. In the recent 5 years, mechanism investigation within multimodal neuroimaging (eg, fMRI, structural MRI, etc) in MMP-induced rehabilitation of SUDs has been lacking. Understanding the diverse neurobiological mechanisms underlying the efficacy of MMPs is crucial for designing more effective paradigms and predicting therapeutic response. Moreover, combined paradigms may further enhance the downregulation of drug craving. For example, in a prospective cohort study of individuals with alcohol disorder, the treatment-as-usual (TAU)+VR CET group showed a greater reduction in alcohol craving compared with the TAU-only group.^15^

## Conclusion

The challenges in preventing relapse during the detox treatment of drug addiction highlight the potential of MMPs as a novel, cost-effective approach for reducing drug craving and identifying the relevant neural biotargets, as shown in table 1. Beyond being an exploratory mechanism and psychological intervention for addictive disorders, MMP may benefit other clinical applications such as surgical treatment (eg, DBS, etc), telemedicine, remote patient care and monitoring. From external cue stimulus to paradigm therapy, MMPs could be used in the entire process of addiction neuroimaging research. While recent studies primarily have focused on the electrophysiological level of the brain, a comprehensive understanding of the multimodal neurobiological mechanisms will enhance the value validation of MMPs that are applied in psychological rehabilitation. MMP could synergise with other paradigms, potentially involving neural mechanisms in plasticity recovery in the reward-punishment circuit, executive function and contextual memory, including the thalamus, hippocampus and frontal regions.^1^ So far, the therapeutic effectiveness of MPP remains unclear, requiring further exploration through comparisons with other routine paradigms and placebo groups. Proposing an optimised approach (eg, MMP, rTMS, TAU, CBT, etc) is essential for maximising the use of medical resources. Therefore, we anticipate that the metaverse will permeate all aspects of clinical practice related to drug addiction, with MMP-induced specific neuroimaging features and therapeutic MMPs accelerating the diagnosis, relapse prediction and tailored treatment of SUD.

**Table 1:** Clinical cases of metaverse medical paradigms applied in the mechanism discovery and relapse prevention of drug addiction in the past 5 years

MMPs	Scenario	Research methodology	Subjects	Significant measurement indexes	Clinical application	Reference
Cue exposure stimulation	8 min social environment video depicting a real-life story of addicts who are taking MA together and who invite the observers to use MA	Cross-sectional study	61 MA abstinent participants vs 45 normal controls	Changes in time domain and non-linear domain of HRV	Monitoring abstinent state via detection of neuro-electrophysiological mechanisms	^7^
	Panoramic videos with 5 min drug-related and 5 min neutral cue	Cohort study	57 MA abstinent participants	Decreased gamma activity in MPFC/OFC and right DLPFC; increased SCL		8
VR-CET	45 min session consisting of six specific smoking-eliciting 3D VR scenes that could interact with participants via varied exposure degrees	Cohort study	39 VR-CET participants vs 32 CBT participants	Lower dropout and relapse rate	Relapse prevention	10
VR-RE	10 min–6h RE training by briefly exposing substance-related stimuli and then obstructing the reconsolidation process of its memories	Cohort study	R-10 min-E MA group vs NR-10 min-E MA group vs R-6h-E MA group vs RV-10 min-E MA group	Measurement of craving, anxiety, HRV and SCL	Possible implications for relapse reduction	9
VRCP	Six 5 min/per VR videos respectively associated with the most common aversive scenarios related to MA-consumption	Cohort study	*Study 1*: 31 VRCP participants vs 29 non-VRCP participants *Study 2*: 612 VRCP participants vs 276 non-VRCP participants	Greater decrease in MA-craving/liking score; greater reduction in HRV indexes on non-linear domain and time domain	Relapse prevention	12
AR-CET	6 smoking-related images and 6 neutral images that are well-integrated into a realistic environment	Cross-sectional study	10 smokers	Higher rates of smoking urge in response to smoking stimuli compared with neutral stimuli	Smoking cessation	14

AR, augmented reality; AR-CET, AR-cue exposure therapy; CBT, cognitive behavioural therapy; 3D, three-dimensional; DLPFC, dorsolateral prefrontal cortex; HRV, heart rate variability; MA, methamphetamine; RV-10 min-E, video cues retrieval-10min interval extinction; NR-10 min-E, VR neutral cues retrieval-10min interval extinction; MMP, metaverse medical paradigm; MPFC/OFC, medial prefrontal cortex/orbitofrontal cortex; R-6h-E, VR cues retrieval-6h interval extinction; R-10min-E, VR cues retrieval-10min interval extinction; SCL, skin conductance level; VR, virtual reality; VR-CET, VR-cue exposure therapy; VRCP, VR counterconditioning producer; VR-RE, VR-retrieval-extinction paradigm.
